# Gonadotropin releasing hormone analogue treatment of central precocious puberty is not associated with altered prevalence of polycystic ovary syndrome: a single center cohort study

**DOI:** 10.1186/s40842-021-00129-4

**Published:** 2021-09-15

**Authors:** Gilad Karavani, Henry H. Chill, Natali Schachter-Safrai, Gan Lomnitz, David Gillis, Dvora Bauman

**Affiliations:** 1 Department of Obstetrics and Gynecology, Hadassah Ein-Kerem Medical Center and Faculty of Medicine, Hebrew University of Jerusalem, Kiryat Hadassah, 91120, PO Box 12000, Jerusalem, Israel; 2 Department of Pediatrics, Hadassah Ein-Kerem Medical Center and Faculty of Medicine, Hebrew University of Jerusalem, Jerusalem, Israel

**Keywords:** Precocious puberty, Gonadotropin releasing hormone analogue, Polycystic ovary syndrome

## Abstract

**Background:**

There is conflicting evidence regarding an association between gonadotropin releasing hormone analogue (GnRHa) therapy and polycystic ovary syndrome (PCOS). This study aimed to compare the prevalence of endocrine disorders, primarily PCOS, between women who had been treated with GnRHa for central precocious puberty (CPP) and those who were not treated.

**Methods:**

This was a retrospective cohort study, including women diagnosed with central precocious puberty between 1989 and 2011 in a university affiliated tertiary medical center. Data collected included demographic data, medical background, clinical presentation at diagnosis and duration of treatment (zero for non-treated). Gynecologic and endocrine long-term outcomes were compared by treatment group.

**Results:**

Fifty-one women were included in the study, 27/51 had been treated with gonadotropin releasing hormone analogue (GnRHa). Overall prevalence of PCOS was 19.6%. No statistically significant difference in prevalence of PCOS was demonstrated between the treated and non-treated groups. Similarly, overall prevalence of either clinical or laboratory hyper-androgenism, was 29.4% and 33.3%, for the treatment and non-treatment groups respectively (*p *= non-significant).

**Conclusions:**

GnRHa treatment for precocious puberty is not associated with increased risk of polycystic ovary syndrome.

## Introduction

Precocious puberty (PP) in females is defined as the appearance of secondary sexual features before the age of 8 years [1, 2]. Central precocious puberty (CPP) is diagnosed when the etiology of precocious puberty is considered to be premature activation of the hypothalamic-pituitary-ovarian axis and is, in females, mostly idiopathic [3–5].

Not all cases of CPP are treated and the decision regarding whether or not to initiate therapy depends upon age, rate of advancement, expected final height, psycho-social factors and child/parent preferences [6]. The major issues associated with CPP are stunting of final height and achievement of pubertal milestones that are outside the child's capacity for psycho-social coping. The severity of these issues and their importance for the individual girl and family determines whether or not therapy is initiated and when it is terminated. Currently, the sole therapy in use for CPP is GnRH analogues, (e.g. triptorelin acetate/embonate (Decapeptyl)) [7, 8].

Data regarding the prevalence of Polycystic Ovary Syndrome (PCOS) in women who had CPP are sparse. Polycystic ovary syndrome is related, among other factors, to high pulsatile secretion of GnRH, which causes higher secretion of luteinizing hormone (LH), lower follicle stimulating hormone (FSH) and increased secretion of androgens, assumed to result in ovulation disturbances [9, 10]. Central precocious puberty is initiated by earlier and higher pulsatile secretion of GnRH occurring at an earlier than normal age thus raising the possibility of a link between PCOS and CPP [9, 11]. Some earlier work demonstrated a higher prevalence of PCOS in females who had precocious puberty [5, 11–13]. However, there is conflicting evidence regarding an association between GnRH analogue therapy and PCOS [14, 15], though these studies either assessed for PCOS only two years after menarche [14] or focused on clinical signs of hyperandrogenism without laboratory or sonographic evaluation for PCOS.

This study aimed to evaluate the prevalence of PCOS as defined by the widespread criteria, and other endocrine outcomes in females diagnosed with CPP according to GnRH analogue treatment, hypothesizing that the GnRH analogue treatment itself may affect its occurrence..

## Methods

We performed a retrospective cohort study at a tertiary university affiliated medical center. Included were patients diagnosed with CPP between 1989 and 2011. Data was collected through a review of patient medical records at time of CPP diagnosis and during follow-up visits at the pediatric endocrinology clinic. All patients included in the study presented with thelarche, pubarche (TANNER score of at least 2 prior to the age of 8 years), or bone age advancement, in addition to an early increase in gonadotropin or alternatively with an early menarche. Diagnosis of CPP was based on evaluation of baseline gonadotropins levels (LH). Biochemical diagnostic criteria for CPP included a serum LH concentration of 5 U/L after GnRH (or leuprolide) administration or a basal LH level of 0.3 U/L using ultra-sensitive assays [5].

Excluded from the study were patients with baseline CNS insults (irradiation, CNS infection or trauma etc.) or pathologies and known endocrinopathies at CPP diagnosis.

We also excluded patients with missing data regarding treatment for CPP or related endocrine and gynecologic disorders at follow-up after 15 years of age. Data collected from medical records included body mass index (BMI) by age, parents' height (measured with a standard wall-mounted stadiometer by the same pediatric endocrinologist (D.G)), past medical treatment or surgeries and presentation at diagnosis. For the pharmacologically treated group of patients, additional information was collected including age at initiation and cessation of treatment, type and duration of treatment, LH levels at treatment initiation and time (months) from cessation of treatment to menarche.

Information regarding follow-up at the age of 15 years or older was collected from the medical records of pediatric endocrinology, general endocrinology and gynecology clinics. Height was measured with accuracy of up to 1 cm and weight of up to 1 kg, BMI (kg /m^2^) is presented as percentile according to age. Androgen hormones levels – dehydroepiandrosterone-sulfate (DHEA-S), androstenedione and total testosterone, were tested according to the normal reference range reported by the laboratory. Laboratory hyper-androgenism was defined by an increase above the upper normal limit in at least one of these hormones.

Clinical hyperandrogenism was defined by documentation of moderate-severe acne, androgenic hirsutism (defined by a Ferriman-Gallwey score above 8) or hair loss (male pattern). Oligo-menorrhea was defined according to menstrual cycle duration of over 35 days. Anovulation was considered in patients with menses more than 60–90 days apart, at least 2 years after menarche. Menorrhagia was defined as total blood loss exceeding 80 mL per cycle or menses longer than 7 days and metrorrhagia as uterine bleeding at irregular intervals, between the expected menstrual periods. Amenorrhea was defined as lack of menarche by 15 years of age or by 3 years after the onset of breast development (primary) or as lack of menses for more than 3 months or 90 days (secondary).

Polycystic ovary definition was according to the sonographic (by either abdominal or vaginal ultrasound) findings in clinical surveillance and in accordance with the practiced criteria [16]. Polycystic ovary syndrome was diagnosed according to the Androgen Excess Society 2006 definition [10] that required the following two parameters: (1) Clinical or laboratory hyperandrogenism and (2) Oligo-menorrhea or sonographic evidence of polycystic ovaries. When evaluating adolescents for PCOS, we used the Diagnostic criteria for polycystic ovary syndrome in adolescents presented by Carmina et al. [17] as these criteria were most commonly used in our institutions' practice.

For all patients in the study data including parameters relevant to PCOS [17] were available for at least 2 years after menarche. In addition, data on the presence of endocrine (including hyperinsulinism, defined as a fasting plasma insulin level higher than 2 μU/mL), metabolic or gynecologic pathologies, as well as obstetric history documented at the follow up, were collected.

### Ethics

The study was approved by the institutional ethical review board (IRB number HMO -192–19).

### Statistical analysis

To test the relationship between categorical variables, the Chi square test or Fisher's exact test were used. Quantitative variables were compared between 2 independent groups using the Student's t-test for normally distributed variables or the Mann–Whitney U test for quantitative variables not normally distributed.

Univariate analysis was performed for factors associated with PCOS in adulthood in for the entire study population and included age, BMI percentile, clinical signs of puberty at CPP diagnosis, LH, FSH and their ratio at CPP diagnosis and treatment with GnRHa. We report odds ratios (OR), 95% confidence interval (CI) for parameters included in the final analysis.

All statistical tests were two-tailed, with a p-value of 0.05 or less considered statistically significant. All statistical calculations were performed using the statistical software package SPSS 24.0 (SPSS Inc., Chicago, IL).

## Results

Fifty-one women were included in the study. Twenty-four had been followed by a pediatric endocrinologist without medical treatment, while 27 had been treated with a GnRH agonist.

Basic parameters did not differ between the groups (Table 1).

**Table 1:** Basic characteristics at diagnosis of central precocious puberty of the gonadotropin releasing hormone (GnRH) analogue treated and untreated groups

Parameter	GnRHa treatment *N* = 27	No treatment *N* = 24	*P* value
Age at diagnosis (years)	7.3 ± 0.6 (5.7–8.0)	7.6 ± 0.6 (6.0–8.0)	0.101
Bone age at diagnosis (years)	9.6 ± 1.7 (7.1–12.0)	10.7 ± 1.7 (7.1–13.0)	0.072
Advanced over chronological age (months)	16.4 ± 11.8 (0–43.0)	21.6 ± 12.0 (8.0–50.0)	0.214
Weight at diagnosis (kg)	32.9 ± 8.6 (22.0–58.0)	36.4 ± 11.7 (17.0–67.0)	0.250
Height at diagnosis (centimeters)	130.6 ± 10.4 (114–149)	135.6 ± 14.2 (98–162)	0.189
BMI percentile at diagnosis	80.5 ± 19.3 (15.2–98.9)	75.7 ± 24.9 (1.0–98.4)	0.480
Medical background			
Neurologic	3 (11.1)	5 (20.8)	0.451
Developmental	2 (8.3)	4 (16.7)	0.402
Fetal			0.175
Small for gestational age	1 (3.7)	4 (16.7)	
Large for gestational age	1 (3.7)	0	
Prematurity (< 37 weeks)	4 (14.8)	1 (4.2)	
Endocrine			0.661
Hypothyroidism	1 (3.7)	0	
NCCAH	2 (7.4)	2 (8.3)	
Vitamin D deficiency	0	2 (8.3)	
Benign premature thelarche	1 (3.7)	1 (4.2)	
Clinical presentation at diagnosis			0.422
Pubarche and thelarche	19 (70.4)	12 (57.1)	
Thelarche only	5 (18.5)	5 (23.8)	
Pubarche only	3 (11.1)	4 (19.1)	
LH at diagnosis (IU/L)	1.9 ± 1.7 (0.1–6.2)	2.1 ± 1.6 (0.3–6.0)	0.722
FSH at diagnosis (IU/L)	3.8 ± 2.3 (1.1–9.4)	4.7 ± 1.9 (1.6–8.3)	0.200
Estradiol at diagnosis (pmol/L)	121.9 ± 56.4 (70–232)	191.2 ± 246.6 (70–1080)	0.296
LH/FSH ratio at diagnosis	0.5 ± 0.3 (0.1–1.0)	0.5 ± 0.4 (0.1–1.4)	0.446

Data presented as Mean ± SD (range) or n (%)

*Note*: *SGA* Small for gestational age, *LGA* Large for gestational age, *LH* Luteinizing Hormone, *FSH* Follicle Stimulating Hormone, *BMI* Body Mass Index, *NCCAH* Non-classical congenital adrenal hyperplasia

The mean age at initiation of treatment was 8.6 ± 1.1 years and treatment was administered for 24.3 ± 12.8 months. Mean age at cessation of GnRHa treatment was 10.8 ± 0.5 years and time from its discontinuation to menarche was 10.0 ± 3.5 months.

Mean follow-up time was 10.9 ± 3.8 years (time range was 5.5–31.8 years, with only one patient followed up more than 16 years). Age at follow up ranged from 15.1–38.8 years. Only one patient was older than 25 years at follow-up. Two patients tried to conceive, both conceived spontaneously within 6 months – one delivered at term and the second had an early spontaneous miscarriage. Age at follow up was similar for the treatment and non-treatment groups (19.9 ± 4.6 vs. 18.7 ± 1.8, respectively; *p *= 0.38). The overall rate of PCOS diagnosis at follow up was 19.6%. Polycystic ovary syndrome occurred at similar rates in the treatment and non-treatment groups (22.2% vs. 12.5%, respectively; *p *= 0.47).

The overall prevalence of either oligomenorrhea or amenorrhea was 33.3%, with similar rates in both groups. Hyperandrogenism, based either on clinical findings or with laboratory evidence, were reported in 29.6% of women in the treatment group and in 29.2% in the non-treated group (*p *= 0.97). The rate of other endocrine pathologies at follow-up was not statistically different between the two groups. (Table 2).

**Table 2:** Comparison of endocrine and gynecologic diagnoses at adulthood

Parameter	GnRHa treatment *N* = 27	No treatment *N* = 24	*P* value
Hypothyroidism	1 (3.7)	1 (4.2)	1.00
Osteoporosis	0	1 (4.2)	0.471
Hyperinsulinism	1 (3.7)	0	1.00
Height (cm)	160.8 ± 4.6 (152–170)	154.8 ± 10.2 (130–170)	0.026
Delta height compared to mid-parental height	-2.8 ± 5.1 (-13-(+ 16.5))	-6.3 ± 7.4 (-24-(+ 2))	0.154
BMI (kg/m^2^)	24.8 ± 5.6 (19.4–40.0)	23.6 ± 3.4 (14.4–30.9)	0.865
PCOS diagnosis^a^	7 (25.9)	3 (12.5)	0.300
Hyper-androgenism^b^	8 (29.6)	7 (29.2)	0.971
Menorrhagia	2 (7.4)	4 (16.7)	0.402
Metrorrhagia	1 (3.7)	2 (8.3)	0.596
Menses			0.671
Regular	17 (63.0)	17 (70.8)	
Oligomenorrhea	8 (29.6)	7 (29.2)	
Amenorrhea	2 (7.4)	0	

Data presented as Mean ± SD or n (%)

*Note*: *PCOS* Polycystic Ovary Syndrome, *BMI* Body Mass Index

^a^ According to the Androgen Excess Society Criteria (2006)

^b^ Either clinical or laboratory based hyperandrogenism diagnosis

We performed univariate analysis for factors associated with PCOS in adulthood in for the entire study population (Table 3). Parameters included age, BMI percentile and clinical signs of puberty at CPP diagnosis as well as LH, FSH and their ratio at CPP diagnosis and whether GnRHa treatment was administered for CPP. None of these parameters were found to be significantly different when comparing CPP patients diagnosed with PCOS and the rest of the cohort.

**Table 3:** Parameters evaluated at central precocious puberty diagnosis and at follow-up according to the later diagnosis of polycystic ovarian syndrome (PCOS)

Parameter	PCOS *N* = 10	No PCOS *N* = 41	*P* value
Age at CPP diagnosis (years)	8.1 ± 1.2 (6.8–10.0)	8.5 ± 1.0 (5.7–10.6)	0.360
Bone age at CPP diagnosis (years)	10.1 ± 1.8 (7.1–12.0)	10.2 ± 1.8 (7.1–13.0)	0.982
Advanced over chronological age (months)	21.0 ± 17.3 (5.0–50.0)	18.7 ± 10.9 (0–43.0)	0.928
BMI percentile at CPP diagnosis	83.5 ± 11.1 (65.0–95.0)	76.8 ± 23.9 (1.0–98.9)	0.988
Fetal			0.151
Appropriate for gestational age	9 (90.0)	36 (87.8)	
Small for gestational age	0	5 (12.2)	
Large for gestational age	1 (10.0)	0	
Prematurity (< 37 weeks)	1 (10.0)	4 (9.8)	1.00
Clinical presentation at CPP diagnosis			0.367
Pubarche and thelarche	5 (50.0)	26 (70.3)	
Thelarche	3 (30.0)	7 (18.9)	
Pubarche	2 (20.0)	4 (10.8)	
LH at CPP diagnosis (IU/L)	2.1 ± 1.9 (0.1–6.0)	2.0 ± 1.5 (0.1–6.2)	0.925
FSH at CPP diagnosis (IU/L)	4.0 ± 2.4 (1.40–8.3)	4.3 ± 2.1 (1.1–9.4)	0.625
Estradiol at CPP diagnosis (pmol/L)	203.3 ± 160.5 (70–518)	150.0 ± 193.7 (70–1080)	0.140
LH/FSH ratio at CPP diagnosis	0.6 ± 0.3 (0.1–1.0)	0.5 ± 0.3 (0.1–1.4)	0.479
GnRH analogue treatment for CPP	6 (60.0)	21 (51.2)	0.735

Data presented as Mean ± SD or n (%)

*Note*: *LH* Luteinizing Hormone, *FSH* Follicle Stimulating Hormone, *BMI* Body Mass Index, *GnRH* Gonadotropin releasing hormone

## Discussion

In this study we focused on women whose puberty was precocious in order to assess a possible association with endocrine sequelae particularly PCOS, in adulthood. Several studies have explored the hypothesis of a shared mechanism underlying the two endocrinopathies. Bridges et al. [18] suggested altered ovarian development attributed to premature exposure to gonadotropins while Esteban et al. demonstrated increased pulsatile LH secretion in precocious puberty persisting into adulthood [19].

PCOS prevalence in an either healthy population varies significantly, ranging from 2.2 to 21.3%, depending on origin, ethnicity and the diagnostic criteria used [20]. The frequency of PCOS in former CPP patients in the present study is in line with previous studies [11–13, 15] assessing a higher PCOS prevalence in this population [11, 15]. Lazar et al. reported a significantly higher frequency of hyperandrogenism in women with a history of CPP compared to a control group of women with no such history [15]. They demonstrated a twofold higher relative risk for development of clinical signs of PCOS in non-treated compared to GnRHa-treated women [15]. They concluded that pubertal suppression might actually reduce the risk of PCOS [15]. In contrast, Chiavaroli and colleagues [13] reported an increased frequency of PCOS in their GnRHa treated group compared to non-treated controls. They hypothesized that GnRHa might worsen insulin resistance associated with puberty and thus predispose to PCOS. In our study however, hyperinsulinism rates were similar in both GnRHa treated and untreated patients and there was no statistically significant difference in the frequency of PCOS between the treated and non-treated groups, possibly due to the small sample size.

Several other endocrinopathies evaluated—hypothyroidism, osteoporosis and hyperinsulinism—did not differ between treated and untreated patients. Though metabolic syndrome and diabetes were not assessed, the similar rate of hyperinsulinism may explain the comparable rates of osteoporosis and hypothyroidism. Hyperinsulinemia is strongly associated with PCOS, and while the pathogenesis is still unclear, other endocrinopathies, such as osteoporosis, have been previously shown to be associated with PCOS through hyperinsulinism [21]. Additionally, thyroid hormones may act as antagonists in the liver so thyroid hormone deficiency may decrease glucose production and increase insulin resistance, suggesting a possible role of hypothyroidism in the pathogenesis of PCOS [22].

Our work, being retrospective, has several limitations including a relatively small sample size, a non-random distribution between treated and non-treated groups and insufficient hormonal and sonographic data relevant to the diagnosis of PCOS. The inclusion of patients diagnosed with PCOS in adolescence and patients with NCCAH, the lack of data regarding race and ethnicity, as well as androgens levels for some of the patients at follow-up are also limitations of this study. Moreover, only six patients were older than 21 years, of which only one patient was older than 25 years. therefore, fertility outcomes were not available for our study population. However, a major strength is the relatively long period of follow-up. Thus, our results strengthen previous findings regarding a possible association between CPP and PCOS.

In conclusion, we did not find any GnRHa treatment-associated difference in frequency of PCOS, two decades and more after the precocious onset of puberty. This data supports the overall safety of GnRHa therapy with regard to PCOS occurrence.

## Data Availability

The datasets used and/or analyzed during the current study are available from the corresponding author on reasonable request.

## Abbreviations

PP: Precocious puberty
CPP: Central precocious puberty
GnRHa: Gonadotropin releasing hormone analogue
PCOS: Polycystic ovary syndrome
